# The Biomarker Revolution in Kidney Transplantation

**DOI:** 10.3390/jcm15197601

**Published:** 2026-09-30

**Authors:** Elizabeth Cho, Yvette Y. Lopez, Sami Alasfar

**Affiliations:** Department of Medicine, Division of Nephrology, Mayo Clinic Arizona, Phoenix, AZ 85054, USA

**Keywords:** transplantation, kidney transplant, allograft rejection, biomarkers

## Abstract

Since the first successful kidney transplant in 1954, transplant medicine has advanced significantly, achieving favorable one-year outcomes. However, despite excellent short-term outcomes, long-term renal allograft survival remains sub-optimal, with acute rejection continuing to be a major contributor to death-censored allograft loss. Current approaches for post-transplant surveillance and rejection monitoring rely on either nonspecific markers that often detect injury late in their course or invasive procedures associated with procedural risks and complications. To address these limitations, numerous non-invasive biomarkers have been introduced into solid organ transplantation research with the goal of improving allograft surveillance, facilitating earlier detection of rejection and complementing existing diagnostic approaches. Although several biomarkers have demonstrated clinical promise, none currently possess sufficient accuracy to replace kidney allograft biopsy. Biomarkers under investigation include blood-, tissue- and urine-based specimens. Their development and validation as independent markers have been limited by smaller sample size, heterogenous targets, variable performance characteristics and suboptimal sensitivity and specificity. In response, the field has increasingly shifted toward multimodal approaches that combine biomarkers and incorporate artificial intelligence to improve diagnostic accuracy and outcomes prediction. This review summarizes current biomarkers, discusses their limitations and explores future directions for biomarker development and clinical implementation.

## 1. Introduction

End-stage kidney disease (ESKD) remains a growing public health concern in the United States, with a prevalence exceeding 800,000 in 2022 according to USRDS data [1]. Approximately 68% of patients with ESKD are maintained on dialysis and 32% receive a kidney transplant, which remains the gold standard treatment, conferring superior survival and quality of life compared with dialysis.

With contemporary immunosuppression strategies, short-term outcomes are excellent, with one-year death-censored graft survival for deceased donor kidney transplants exceeding 95% in the United States [2,3]. Long-term allograft survival, however, remains sub-optimal with, in one contemporary single-center cohort study, allograft survival rates at 5, 10 and 20 years of approximately 80%, 60.6% and 33.3%, respectively [4], and reportedly approximately 40% of transplant failing at 10 years [5]. Acute rejection is a notable cause of death-censored allograft loss, affecting up to 40% of transplant patients [6].

Currently, the standard of care for post-transplant monitoring and surveillance for allograft rejection includes serum creatinine, metabolic or renal function panels, donor-specific antibodies (DSAs), urinalysis, viral testing and kidney allograft ultrasound to exclude other causes of acute kidney injury. To date, however, the gold standard for the diagnosis of kidney allograft rejection remains tissue biopsy interpreted according to the Banff classification. These conventional monitoring protocols have important limitations. Serum creatinine is a late, non-specific marker of allograft injury; it is dependent on muscle mass, insensitive to chronic and subclinical changes, with levels often rising only after significant histologic injury [7,8]. DSA detection is also imperfect, as de novo DSA with stable allograft function is only approximately 50% predictive of subclinical rejection [9].

Although kidney biopsy remains the diagnostic gold standard for allograft rejection, it is invasive and carries procedural risks such as bleeding, arteriovenous fistula formation, and sampling variability with wide inter-observer variability in interpretation among renal pathologists [10,11]. Although the Banff Classification provides a standardized framework for allograft rejection diagnosis, interpretation remains subject to observer variability. Additionally, there is no universal standardization of renal allograft biopsies practice. Protocols for surveillance versus for-cause biopsies are established independently by each transplant center, resulting in further variability in biopsy indication, timing and frequency. Collectively, these limitations make consistent analysis and comprehensive research in allograft rejection challenging and underscore the need for objective and reproducible non-invasive tests and protocols that can accurately diagnose rejection, facilitate timely treatment and ultimately improve long-term allograft survival.

In recent years numerous invasive and non-invasive biomarkers have been introduced into solid organ transplantation research with the goal of clinical implementation. This review addresses current biomarkers, their limitations and future directions for kidney transplantation research (Figure 1).

## 2. Blood Based Biomarkers

### 2.1. Donor-Derived Cell Free DNA

Cell free DNA has been incorporated into several subspecialities such as obstetrics and oncology since its discovery in 1948 by Mandel and Metais. These are small fragments of double stranded DNA, 50–200 base pairs, released into circulation, plasma, urine and other extracellular fluids, by a variety of cells via active secretion or as a response to stress events such as apoptosis or necrosis. The half-life of circulating cell free DNA is short, lasting between 16 min and 2.5 h [12], making it an ideal target to identify acute changes in the host environment due to injury or disease.

The application of cell free DNA in transplantation has gained traction as the ability to differentiate between “self” and “non-self” has been refined. Identification of the genetic differences between donor and recipient has allowed cell-free DNA to expand into the field of transplantation, leading to donor-derived cell-free DNA (dd-cfDNA). In transplantation, dd-cfDNA is reported as both an absolute value and fraction [13]. Numerous studies have evaluated the interpretation of results and threshold values of the absolute vs. fraction of dd-cfDNA, however it was the DART study that established the standardization of the threshold value of detection at ≥1% to distinguish between rejection and no rejection, with an AUC of 0.74 and a positive predictive value of 61% and a negative predictive value of 84% for any rejection and 96% for antibody mediated rejection (AMR) [13]. This was supported by the ADMIRAL study, a large multicenter longitudinal observational cohort study, in which a cut-off of >0.5% correlated with clinical and subclinical acute rejection versus a no-rejection phenotype [14]. Furthermore, in the TRIFECTA study, dd-cfDNA was compared to allograft biopsies and molecular data, MMDx™, to identify acute rejection (TCMR and AMR). Elevations in dd-cfDNA were found to be correlated with allograft rejection, strongest with AMR, allograft injury and inflammation [15]. To date, utilization of dd-cfDNA in clinical practice has gained momentum given its significant NPV to rule out acute AMR; however, incorporation into practice remains inconsistent across institutions, in regard to indication and timing—similar to current limitations of renal allograft biopsies.

Although dd-cfDNA is a promising biomarker, its principal limitation is the inability to distinguish rejection from other forms of allograft injury or nonspecific inflammation. Additionally, this test in isolation falls short of differentiating between AMR and TCMR, which has significant treatment implications. Another limitation of dd-cfDNA testing is the timing of the collection relative to transplant and biopsy. Additionally, dd-cfDNA testing assays are processed through centralized laboratories, potentially delaying clinical decision-making in patients with suspected allograft rejection and thus, contributing to long-term allograft failure. Interpretation of dd-cfDNA in the setting of multiorgan or retransplants is still unknown and requires additional studies.

### 2.2. Gene Expression Profiling

Another upcoming blood-based biomarker is gene expression profiling (GEP). This is based on the molecular framework, transcription of DNA into RNA, which is then translated into proteins under the influence of specific microenvironments. Cells express or repress a combination of genes specific to a healthy or diseased state. The activation and/or suppression of a subset of genes gives rise to GEP (Figure 2).

Based on this phenomenon, immunologic and inflammatory profiles were established as a potential biomarker for kidney allograft rejection. The first GEP assay, TruGraf^®^, was approved by CMS and validated in the Clinical Trials in Organ Transplantation 08 (CTOT-08), a multicenter observational study. Friedwald et al. evaluated 307 patients with surveillance and for-cause biopsies paired with a TruGraf^®^ test result over the course of 24 months. TruGraf^®^ was shown to have an NPV of 78–88% for subclinical acute rejection and a PPV of 47–61% [16]. Interestingly, this study highlighted that a subset of follow-up biopsies after treatment for rejection correlated with a persistently abnormal test, “not transplant excellence” (Not-Tx). This was suggestive of ongoing rejection. A follow-up study by Heilman et al., a prospective multicenter observational study with 240 kidney transplant recipients, compared the transplant excellence (Tx) group to a group with >1 Not-Tx test result. The >1 Not-Tx group had lower eGFR, more chronic changes on biopsy, and overall inferior graft survival [17]. Multiple test results of Not-Tx in the first year of kidney transplantation suggests ongoing alloimmune activation thus increasing the risk of long-term allograft loss. Of note, TruGraf^®^ is indicated as a surveillance tool, measured in patients with a stable allograft function to assess immune status.

Similar to dd-cfDNA, GEP lacks specificity for differentiating rejection from other inflammatory or injury-related processes and remains insufficient for reliably distinguishing TCMR from AMR. There are limited studies evaluating GEP in multiorgan and retransplant patients. The current GEP assays also cannot differentiate between acute kidney inflammation and injury from rejection, AMR or TMCR. Another limitation is the utilization of GEP testing in patients with chronic or progressive allograft dysfunction. The majority of the studies available investigated GEP in acute or subclinical rejection and infections. Indication and interpretation of testing in chronic or chronic active AMR remains unknown. Replication and external validation of GEP assays have been inconsistent. For example, kSORT, a 17-gene expression assay by Immucor Inc., was initially validated in the AART trial, a case–control study across eight transplant centers, including 558 blood samples assessed by quantitative RT-PCR in one center. The gene panel was selected based on analysis from 143 samples and validated in a separate cohort of 124 independent samples. The gene set was then evaluated to detect acute rejection in 191 serial samples; acute rejection was detected up to 3 months prior to detection on renal allograft biopsies [18]. A follow-up multicenter retrospective study across four transplant centers with 1763 blood samples failed to replicate previously published kSORT validity. Acute rejection was reported in 10.7% of the blood samples and 34.8% for any rejection, (including borderline changes) [19]. The performance inconsistencies to detect rejection could be attributed to the larger sample size, variability of disease phenotypes to which transplant patients are susceptible, institutional variations in indication and timing of biopsies to which GEP testing was compared. Additional large-scale studies are required to define patterns of gene expression to differentiate between infection, injury, TCMR and AMR.

### 2.3. microRNA

Another approach to investigating blood-based biomarkers was microRNAs (miRNAs). MiRNA are regulators of gene expression and are integral in the maintenance of homeostasis and disease pathogenesis. First discovered in 1993, they are small (20–22 nucleotides long), non-coding, single stranded RNA that regulate the development, maturation and function of protein coding genes by translational repression and mRNA degradation. MiRNA are stable in extracellular fluids and can be isolated from blood, urine, saliva, plasma and other bodily fluids [20]. They are also tissue- and cell-type-specific. More than 2,300 true mature human specific miRNAs have been identified, approximately 1100 of which have been implicated in human diseases ranging from cardiovascular disease, infection/sepsis, cancer and now renal disease [21,22].

Previous studies exemplified alterations in mRNA expression in an acute rejection episode, most notably genes involved in cell cycling, metabolism and immunity [22]. The landmark study by Anglicheau et al. identified 365 mature human miRNAs from renal allograft biopsies using TaqMan Low Density Array (TLDA). The initial training set included the 365 mature human miRNAs in seven kidney transplant biopsies (4 normal and 3 acute rejection), and were then validated in an individual set of 26 biopsies (17 normal and 9 acute rejection). The following miRNAs were found be overexpressed in acute rejection biopsies and peripheral blood mononuclear cells: miR-142-5p, -155 and -223, while miR-10b, -30a-3p and let-7c were under-expressed. They also found that intragraft expression of miR-142-5p or miR-155 was able to predict acute rejection and allograft status accurately [23].

Matz et al. subsequently identified a panel of five miRNAs to differentiate severe vascular rejection (Banff 4-II/III) from stable renal graft function: miRNA-15B, -16, -103A, -106A and –107. A total of 286 miRNAs was significantly expressed in at least one microassay which was narrowed down to 23 potential candidates after hierarchical clustering. The final combination of the five miRNAs had a high median expression difference in the microarray analysis and the select set was validated in the same sample study. In the multivariate analysis, the combination of the five final miRNAs had an AUC of 0.97 with 100% sensitivity and 90% specificity to differentiate stable graft function from TCMR [24]. This study, compared to previous studies included a sample with urinary tract infections, albeit small, it helps delineate miRNAs expressed during an episode of infection from rejection, potentially adding more weight to the validity of the miRNAs isolated as a potential biomarker for allograft rejection. The study highlights the inability to distinguish rejection from interstitial fibrosis and tubular atrophy, limiting its clinical utility.

miRNA profiling remains a promising area of investigation, however the list of potential miRNAs is large, making the identification of individual miRNAs or a defined panel difficult. In the study by Anglicheau et al., over 300 potential miRNAs were first isolated and Matz et al., identified 23. Consistency across large sample sizes, from similar sources (tissue, blood, urine, other body fluid) and across varying disease phenotypes are still needed. Validation studies of gene expression patterns across disease states such as acute interstitial nephritis, urinary tract infections, viral infections, TCMR and AMR are still lacking. Urinary miRNAs has also been investigated but, again, were inconsistent across various studies as discussed below.

### 2.4. Torque Teno Virus

Torque Teno Virus (TTV) is a small (3.5–3.9 kb), non-enveloped, single-stranded, circular DNA virus. It was first described in 1997 in a case report of a virus associated with post-transfusion hepatitis of unknown etiology. Later in 2002, it was revised as a nonpathogenic virus that is inherent to the human virome and present in up to 98% of the general population and an estimated 99% in the post-transplant population [25,26]. TTV can be measured in whole blood, serum, plasma and urine; due to its inherent property and ease of measurability, it has emerged as a gauge of immune system performance. Within transplantation, TTV load has been shown to be strongly associated with the degree of immunosuppression (Figure 3).

TTV was phased into solid organ transplantation as a unique biomarker looking at the net state of the immune system as opposed to inflammatory markers—high levels indicated over-immunosuppression and increased risk of infection, while lower levels indicated under-immunosuppression [25]. Fluctuations of these levels have previously been studied in various infectious states and tumor propagation. This has since been translated into transplantation as an indirect assessment of immunosuppression since the 2000s, to identify the middle ground between the risk of allograft rejection and infection.

The prospective observational, TTV-POET trial evaluated the risk of allograft rejection and infection using TTV levels and suggested an optimal TTV range to reduce the risk of rejection and infection in the first year post-transplant. The study enrolled 386 kidney transplant recipients with TTV monitoring for 12 months post-transplant. The three-month biopsy was analyzed with TTV load for rejection and infection. In the rejection and infection risk analysis, the odds for allograft rejection decreased by 22% with each log level increase in TTV and the risk of infection increased by 11% with every log increase in TTV. The investigators proposed a target TTV range of 1 × 10^6^ to 1 × 10^8^ c/mL to guide immunosuppression therapy and balance allograft rejection and infection [27]. Further studies investigated the TTV viral load and risk of opportunistic viral infections, such as CMV, EBV and BK. Rosiewicz et al. looked at the correlation between TTV, coinfection with opportunistic viral infections and, the effect on renal allograft function in 93 kidney transplant recipients. The incidence of TTV was 100%, BK 28%, CMV 26.9%, EBV 7.5%, HHV-6A 51.6%, UTI 28% and other bacterial infection 15.1%. Coinfection of TTV/BK and TTV/HHV-6A was most frequent but not associated with a significant change in eGFR at 12 months. The authors also noted earlier occurrence and higher incidence of BK reaction with lower TTV loads [28].

TTV has shown great promise in the personalization of post-transplant care with limitations mainly due to small sample sizes. Larger studies are still necessary to validate testing and determine how to incorporate it into clinical practice. The TTV R-Gene^®^ assay distributed by Biomérieux is the most used assay at this time, although there are clinical trials now underway, utilizing a decentralized system. The shift in testing presents its own challenges in regard to consistency and longitudinal application. Independent TTV assays introduces challenges to comparisons across institutions and long-term follow-up. Intrapersonal variability also makes standardization inherently difficult, requiring monitoring of personalized trajectories over time, which can be a significant barrier in decentralized testing.
**Blood-Based Biomarker Summary**Non-invasive, precision-focused for surveillance and early detection of allograft injury/rejectionConfounded by non-specific inflammation, infection, and tissue injuryLimited validation due to low rejection rates (≈10–15%), small event numbers, and challenges with reproducibility across cohortsCostly: Approximately $1300–3000 per assay; many studies rely on serial testing, increasing overall expense [29,30] *Rapidly evolving field with expanding biomarker platforms (dd-cfDNA, gene expression, miRNAs, proteomics) and ongoing refinement of algorithmsCurrent role: as an adjunct to clinical assessment and biopsy; implementation remains institution-dependent and not a replacement for tissue diagnosis* Reported cost of miRNAs and TTV assays is not available; the estimate of testing costs is approximated by dd-cfDNA and gene expression profiling testing.


## 3. Urine Based Biomarkers

### 3.1. CXCL9, CXCL10

Urinary cellular miRNAs and urinary chemokines emerged as promising biomarkers for kidney transplant monitoring, primarily because they offer a noninvasive alternative to biopsy for detecting allograft rejection and inflammation. This approach is particularly attractive given the relatively low risks, cost, and patient burden as compared to allograft biopsies.

Among these biomarkers, the interferon gamma–induced chemokines C-X-C motif ligand 9 (CXCL9) and C-X-C motif ligand 10 (CXCL10) have been the most extensively studied. These molecules are secreted by infiltrating immune cells as well as renal tubular and mesangial cells, and their urinary levels correlate with both tubulointerstitial and microvascular inflammation in kidney allografts. Early work by Jackson et al. demonstrated that urinary CXCL9 and CXCL10 were significantly elevated during episodes of acute rejection and not in recipients with stable allograft function, CNI toxicity or IFTA supporting their role as indicators of immune activation [31].

Subsequent studies reinforced their potential clinical utility. Urinary CXCL9 and CXCL10 have been shown to reflect immune activity and may help predict acute rejection. For example, CXCL10 normalized to creatinine (CXCL10:Cr) demonstrated reasonable diagnostic performance, detecting TCMR with an AUROC of 0.80 and AMR with an AUROC of 0.76, even in cases lacking clear tubulointerstitial inflammation (AUROC 0.70). Importantly, combining CXCL10:Cr with DSA levels further improved diagnostic accuracy for AMR (AUROC 0.83) and helped stratify graft loss risk, highlighting the value of integrating urinary biomarkers with immunologic data [32]. More recently, Van Loon et al. developed an integrated model combining urinary chemokines with clinical parameters, which showed additional promise and reported an AUROC of 81.3% for acute rejection detection and suggested that such approaches could significantly reduce the need for protocol biopsies [33].

Additionally, urinary chemokines have shown variable clinical performance. A large 2025 multicenter prospective study involving 733 kidney transplant recipients reported only modest diagnostic accuracy, with an AUROC of 0.70 for CXCL9 and 0.64 for CXCL10 in detecting allograft rejection [34]. These findings suggest that CXCL9 has limited utility, and that CXCL10 adds minimal diagnostic value.

Despite these advantages and advances, several limitations have become apparent overall. A major challenge is a lack of specificity, as urinary CXCL9 and CXCL10 are also elevated in other inflammatory conditions, particularly BK viral infection and urinary tract infections. Early studies already showed comparable elevations in both rejection and BK virus–associated inflammation [31]. This overlap complicates interpretation and reduces their reliability as standalone diagnostic tools. Another key limitation is their inability to distinguish rejection subtypes. Differentiating TCMR from AMR remains difficult when relying solely on urinary chemokine levels, necessitating adjunctive markers such as DSA for improved classification.

Finally, confounding factors and overlapping injury signals further complicate interpretation. Urinary chemokines are elevated in multiple causes of kidney injury, including infections and nonimmune damage. Other urinary biomarkers—such as TNF, NGAL, kidney injury molecule-1 (KIM-1), liver-type fatty acid–binding protein (L-FABP), and N-acetyl-β-D-glucosaminidase (NAG)—reflect tubular injury but lack specificity for rejection. For example, NGAL is markedly elevated in acute tubular necrosis (ATN), often reaching levels approximately tenfold higher than in acute rejection (AUROC 0.93 for ATN diagnosis), while KIM-1, L-FABP, and NAG indicate proximal tubular damage regardless of etiology [35,36].

### 3.2. Urinary Cell mRNA (Like CD3ε, Perforin, Granzyme B)

Urinary-cell mRNA molecules, isolated from immune and epithelial cells shed into the urine of kidney transplant recipients, providing noninvasive biomarkers that reflect immunological activity within the allograft. This approach has been shown to capture immune activation within the allograft. Early studies demonstrated that perforin and granzyme B mRNAs, key effectors of cytotoxic T cells, are elevated during rejection in urine [36,37]. Subsequent work expanded these analyses to multi-gene panels, showing that urinary cell mRNA profiles can predict TCMR and distinguish graft injury from stable function [38,39].

The multicenter Clinical Trials in Organ Transplantation-04 (CTOT-04) study validated a 3 gene urinary mRNA signature comprising CD3ε, IP-10 (CXCL10), and 18S ribosomal RNA, which noninvasively detects acute cellular rejection [40]. This panel achieved an AUC of 0.85, demonstrating high sensitivity and specificity, 79% and 78% respectively, for distinguishing acute cellular rejection from stable graft function. Importantly, urinary transcript levels rose prior to biopsy-confirmed rejection, supporting their use for early detection and longitudinal monitoring [40].

Similarly to urinary chemokines/cytokines, elevated urinary mRNAs can also occur in urinary tract infections, BK virus nephropathy, and other inflammatory states, limiting specificity [36,39,40]. Additionally, urine mRNA was more informative for detecting a T-cell–mediated rejection, with less utility for antibody-mediated rejection (AMR) or chronic injury/fibrosis [40].

### 3.3. Urinary Exosomal mRNA

Urinary exosomal mRNAs are another emerging noninvasive biomarker for kidney transplant monitoring. Exosomes are small extracellular vesicles secreted by renal and immune cells that carry stable RNA, including mRNA and microRNA, which reflect allograft immune activity. Because exosomal RNA is protected from degradation, it allows for more robust detection than total urinary RNA. Recent studies have shown that multi-gene exosomal mRNA signatures can distinguish acute rejection from stable graft function with high accuracy, achieving an AUROC of 0.93 [41]. Exosomal biomarkers are enriched for graft-specific transcripts, enabling earlier detection of T-cell–mediated rejection, improving reproducibility, and offering the potential to distinguish rejection subtypes and monitor therapy.

Exosomal mRNA profiling is limited by technical complexity, higher cost, and incomplete validation in large cohorts, and its specificity can be reduced by infections or other inflammatory conditions. Despite these challenges, combining exosomal mRNAs with urinary chemokines and cellular mRNA panels offers a comprehensive, noninvasive approach for real-time kidney graft monitoring.
**Urine-Based Biomarker Summary**Non-invasive, low-cost approach for real-time surveillance of allograft inflammation and rejectionConfounded by other inflammatory conditions, particularly BK virus nephropathyTechnical limitations: sample-processing variability, mRNA instability/degradation, and inability to fully distinguish renal allograft from urogenital tract signalsCost-effective: approximately $200 per assay [42]; biomarker panels may increase costs but provide broader biologic insightCurrent role: promising screening and surveillance tool

## 4. Tissue Based Biomarkers

### MMDX™ & HistoMAP™ Kidney

Direct tissue evaluation via allograft biopsy based on the Banff Classification remains the gold standard to diagnose rejection. There have been many iterations of the Banff Classification since its first consensus statement in 1991 to define and classify renal allograft rejections; however, limitations persist to this day: namely, the variability of the experience of the reading pathologist and sampling biases. Investigations into the molecular patterns directly extracted from graft tissue were introduced with MMDx™ and Histomap™ Kidney. These tests were developed to improve diagnostic utility and delineate rejection phenotypes.

MMDx™ was developed in a series of stages based on a large-scale peer-reviewed grant, the Genome Canada project. It is a microarray-based assay that analyzes the molecular footprints for disease archetypes directly from tissue. MMDx™ measures the expression of 19,462 unique genes in a biopsy sample. The sample was then processed by a machine learning algorithm against an established reference set of well-defined biopsies to classify the sample biopsy into a disease archetype. The archetypes include: TCMR, AMR, acute kidney injury and atrophy-fibrosis. The INTERCOMEX study showed the concordance rate between MMDx™ and histological diagnosis was 77% for TCMR, 77% for AMR and 76% for no rejection [43]. Further investigations looked into the discrepancy between histologic diagnosis and MMDx™ testing. Madill-Thomsen et al. reported a discrepancy rate of 37% between the MMDx™ final report and biopsy (AMR was 22%, TCMR was 61%, and no rejection was 24%) [44]. Histomap™ Kidney, is another tissue-based molecular test manufactured and distributed by CareDx, Inc, available for research purposes only at this time. HistoMAP ™ serves as an adjunct to histopathology to diagnose allograft rejection by phenotype [45]. The purpose of HistoMAP™ Kidney is to complement histological diagnosis, thus further eliminating uncertainty in renal allograft rejection. It is currently only available through a clinical trial that initiated in 2026.

Despite its molecular approach, MMDx™ remains dependent on biopsy tissue and therefore shares limitations related to its sampling variability. Discrepancies in the MMDx™ and histological interpretation require a second look at the histopathology, thus not completely eliminating the need for an invasive procedure and interpretation by a renal pathologist. The incremental value of molecular classification beyond expert histopathologic assessment remains an area of active investigation.
**Tissue Based Biomarker Summary**Objective, precision-based assessment; useful in ambiguous casesConfounded by non-rejection inflammation & tissue injuryInvasive and prone to sampling errorCostly: ~$3000 + central lab processing [30]MMDx™ may improve as datasets and algorithms evolve; HistoMAP™ only available in clinical trialsCurrent role: serves as an adjunct to histology, not a replacement

## 5. Challenges in Biomarker Validation and Clinical Implementation

Despite substantial advancements in biomarker discovery, translation into routine clinical practice has remained limited (Table 1). A major challenge is the considerable heterogeneity among studies evaluating kidney transplant biomarkers. Differences in patient populations, timing of transplantation, immunosuppressive regimens, biopsy indications (surveillance vs. for-cause) and the evolving Banff classification criteria make direct comparisons between studies difficult. Additionally, rejection is not a single disease entity but is a spectrum of phenotypes—TCMR, AMR, mixed, borderline, microvascular inflammation, etc., each with distinct pathophysiologic patterns and management strategies. As a result biomarkers that perform well in one cohort may not demonstrate the same diagnostic accuracy in another. Furthermore, cost, assay accessibility, reimbursement variability and integration into clinical workflows remain substantial barriers to widespread adoption.

Validation of biomarkers also complicates interpretation of tests. Many assays are performed using different platforms, lab protocols, thresholds and reference standards, limiting reproducibility across centers, especially as assays are decentralized such as with urinary biomarkers. Additionally, many studies are observational and include relatively small numbers of rejection events, which can overestimate diagnostic performance and limit external validation.

Currently, many of the biomarkers available fall short of distinguishing between allograft rejection and nonspecific injury. Consequently, positive results may be observed in cases of infection, ischemia–reperfusion injury, drug toxicity, or chronic allograft injury. This overlap reduces specificity and contributes to the persistent need for histopathological confirmation.

## 6. Multimodal Approaches

### 6.1. iBox

The search for individual non-invasive biomarkers to detect acute rejection is still underway. Individually, the tests listed above have great potential but continue to face limitations, mainly the inability to discern inflammation from injury. The need for non-invasive biomarkers to diagnose rejection is critically important, as these tools impact long-term allograft survival. To fill this gap, the Paris Transplant Group proposed Integrative Box (iBox) technology, a prediction system to assess the risk of allograft loss in transplant recipients. iBox gathers data from recipients at any time point post-transplant and generates the probability of graft loss up to 7 years. The metrics included in the prediction tool include the following: eGFR, proteinuria, presence of DSA, and kidney allograft biopsy (activity and chronicity scores). In the initial study by Loupy et al., the derivation cohort included 4000 consecutive kidney recipients across four French centers. From the derivation study, iBox showed a C-index of 0.81. The validation cohort included 2129 kidney recipients from three European centers and 1428 kidney recipients from North American centers. The C-index in the European and North American cohorts was 0.81 and 0.80, respectively [46]. iBox was shown to have high discrimination for death-censored graft loss across varying patient populations (donor types, racial groups, immunosuppression regimens). The iBox scoring system received qualification as a secondary efficacy endpoint in 2022 and was subsequently recognized within the FDA Biomarker Qualification Program as a reasonably likely surrogate endpoint for kidney transplant clinical trials [47].

### 6.2. GEP and dd-cfDNA

With the current limitations of individual biomarkers, there has been a shift to further investigate if a combination of testing is a superior strategy to identify allograft rejection. Previous studies established that GEP was better at detecting ACR than dd-cfDNA, with an AUC-ROC of 0.8 and 0.62, respectively. For AMR, dd-cfDNA outperformed GEP with an AUC-ROC of 0.84 vs. 0.71, respectively [48]. In the post hoc analyses from the CTOT-08 study, the diagnostic performance of the combination of GEP and dd-cfDNA was investigated to detect acute TMCR and subclinical AMR. A total of 428 samples collected from 208 patients were evaluated, of which 325 were no rejection and 103 were subclinical rejection. Results showed that when both assays were negative, the NPV was 88%. When both assays were positive, the specificity was 98% with a PPV of 81%. The combination of two tests improved the AUC-ROC to 0.81, which was significantly greater than each individual test, GEP of 0.75 and dd-cfDNA of 0.72 [16]. Unfortunately, follow-up studies failed to reproduce these results. In a multicenter study in Spain, 230 consecutive kidney transplants with for-cause biopsies had paired testing with TruGraf^®^ and TRAC™. The AUC-ROC was <0.65 for individual or combined biomarkers to accurately diagnose rejection [49]. This is consistent with previous results: a negative test carries significant weight for no rejection, however positive results or discrepancies between GEP and dd-cfDNA can indicate a wide range of pathology, from subclinical acute ACR, early AMR or AMR, thus prompting invasive testing with a renal allograft biopsy.

### 6.3. Tutivia

Tutivia is a peripheral GEP assay that includes a 17-gene mRNA signatures and is unique because it incorporates an artificial intelligence (AI) algorithm to stratify patients into low or high risk categories for acute rejection. Tutivia was first established using the clinical samples from the Genomics of Chronic Allograft Rejection (GoCAR) Study as the training set, to predict subclinical/borderline acute cellular rejection and identify recipients at high risk of acute rejection and allograft loss. Tutivia was then validated in a non-randomized, prospective, global observational study (NCT04727788) with histopathology results based on the Banff 2019 criteria. The validation study included 151 kidney transplant recipients from five countries, 71% of biopsies were surveillance or protocol biopsies and 29% were for-cause biopsies. The AUC for Tutivia was 0.69, compared to creatinine alone at time of biopsy, which had an AUC of 0.51. Using a cut-off of 50 (based on a scale of 0–100), Tutivia categorized patients into low vs. high risk of rejection, with an NPV of 79% and a PPV of 60% [50]. Interestingly, when evaluating Tutivia in biopsies positive for BK virus, SV40 was highly correlated with a low-risk Tutivia score. Overall, this test has the potential to identify or risk stratify early acute rejection in kidney transplant recipients; however, it is not reliable as a standalone test. Extensive validation studies are still needed across larger cohorts that also include subset analyses for viral infections.

### 6.4. BIOMARGIN

One of the most comprehensive collaboratives is currently underway in Europe to discover and validate noninvasive biomarkers (mRNAs, miRNAs, peptides, proteins, lipids and metabolites) in renal transplantation for allograft rejection and allograft injury. BIOMARGIN (Biomarkers of Renal Graft Injuries) is a stepwise investigation incorporating a combination of blood and urine biomarkers. The first phase is the discovery of a “training set” which will result in a list of biomarker candidates. Next is the identification of a “selection set” of biomarkers. The “selection set” is then validated through trans-sectional studies. Finally, the validated biomarkers will undergo diagnostic and prognostic evaluations to reach a consensus on the final set of biomarkers. Throughout the process, the biomarkers will be compared against blinded histological analysis by expert pathologists. Blood and urine samples will be obtained from four large biobanks: CHU Limoges, MHH, KU Leuven, and AP-HP. To date, the program has collected over 2500 biopsies, 4200 plasma samples, and 9700 urine samples from 1300 patients. This is one of the most comprehensive bench-to-bedside discovery and validation initiatives for tools to predict graft lesions and predict renal function outcomes [51].

## 7. Future Directions

Transplant medicine has evolved significantly over the past few decades, leading to significant improvements in both patient and allograft survival. Despite these advances, there remains a critical need for accurate diagnostic and prognostic tools that enable early detection of allograft rejection. Although numerous biomarkers have emerged as promising candidates, no single test has demonstrated sufficient accuracy and reliability to replace the allograft biopsy. As a result, the attention has shifted toward multimodal strategies that integrate complementary biomarkers, each capturing different aspects of the alloimmune response [52]. This approach has the potential to provide a more compressive assessment of allograft health, improve diagnostic precision and support timely treatment decisions. Ultimately, combining multiple biomarkers may reduce reliance on invasive procedures, minimize diagnostic uncertainty, and facilitate more personalized post-transplant management.

The integration of AI has begun to permeate various fields including medical research. AI is increasingly being incorporated into transplantation research, particularly in molecular diagnostics, digital pathology, biomarker integration and outcome prediction. AI-based methods may facilitate the identification of complex biological patterns that are not readily detectable using conventional statistical approaches. Recent reviews have highlighted the potential for AI to enhance transplant diagnostics, optimize immunosuppression strategies, improve risk prediction and integrate multimodal data for complex clinical decision-making. However, widespread clinical adoption requires stringent and rigorous validation, standardization and oversight to ensure reliability and generalizability [53]. Despite the promise of AI-based models and algorithms, challenges related to external validation, algorithmic bias, model transparency, data governance and integration into clinical workflows remain significant barriers to implementation [54] MMDx™ is one such example of AI with a machine learning algorithm incorporating GEP. AI can be utilized to analyze and optimize a panel of tests (blood-, urine-, and tissue-based) into a step-wise algorithm to stratify disease phenotypes thus driving management decisions and affecting long-term outcomes. AI in histopathology is also being explored [55]. One of the consistent limitations of renal allograft biopsies interpretation is interobserver variability despite the Banff Classification. Farris et al. have demonstrated how AI may enable more quantitative, objective and reproducible assessment of kidney transplant histopathology, with ongoing efforts toward automated Banff classification systems [56]. Risk prediction models are also increasingly leveraging machine-learning approaches, for example, the iBox model that integrates clinical, immunologic, and histologic variables to predict long-term allograft outcomes and support risk stratification [46].

Further predictive platforms may integrate molecular biomarkers, clinical variables, histopathology, genomics and social determinants of health to enable more precise risk stratification and individualized immunosuppression strategies. Ultimately, the future of transplantation can extend beyond the diagnosis of allograft rejection, toward optimizing recipient–donor matching, personalizing immunosuppression and guiding long-term post-transplant management.

The emergence of AI has substantial benefits, but precautions must be taken to protect patient information. Contemporary models have the ability to process large volumes of sensitive, multimodal patient data, introducing vulnerability to breaches even with de-identified data [57]. As AI increasingly crosses borders, protecting cross-national data sharing and delineating jurisdictional authority will be essential.

## 8. Conclusions

The drive to develop non-invasive biomarker research in kidney transplantation stems from the persistent challenge of suboptimal long-term kidney allograft survival. While early detection of allograft injury is essential, accurate identification of the underlying injury phenotype is equally important because treatment strategies vary substantially and directly influence long-term graft outcomes. Since the first successful kidney transplant in 1954, remarkable advancements have been made in donor–recipient matching, immunology, induction therapies and maintenance immunosuppression. Despite extensive efforts to identify individual biomarkers or biomarker panels, current approaches have not reliably distinguished specific rejection phenotypes from non-immune-mediated inflammation or injury with sufficient confidence. Although some biomarker assays, such as dd-cfDNA, demonstrate high NPV and may be useful for ruling out rejection, these findings should be interpreted cautiously, as their performance may be influenced by the relatively low prevalence of rejection in study populations. Consequently, available biomarkers primarily serve as adjuncts to, rather than replacements for, the allograft biopsy. These limitations are not unique to kidney transplantation. Lessons from biomarker development in chronic kidney disease suggest that successful clinical implementation requires more than statistical association with outcomes. Biomarkers must demonstrate reproducibility across diverse populations, incremental value beyond established clinical testing and meaningful impact on clinical decision-making [58]. The experience with cardiovascular disease biomarkers in CKD underscores the importance of large-scale validation studies and standardized development frameworks before widespread adoption in transplantation. Further breakthroughs in transplant diagnostics will likely depend on the integration of AI and large-scale multicenter datasets, enabling more sophisticated biomarker discovery, improved validation across diverse populations and ultimately more precise, noninvasive assessment of allograft health. Although no biomarker currently demonstrates sufficient diagnostic accuracy to replace allograft biopsy, accumulating evidence suggests that multimodal approaches integrating complementary biomarkers with AI may substantially improve the detection, classification and management of allograft injury. Continued validation through large multicenter studies will be critical before these technologies can be broadly implemented in routine clinical practice.

## Figures and Tables

**Figure 1:**
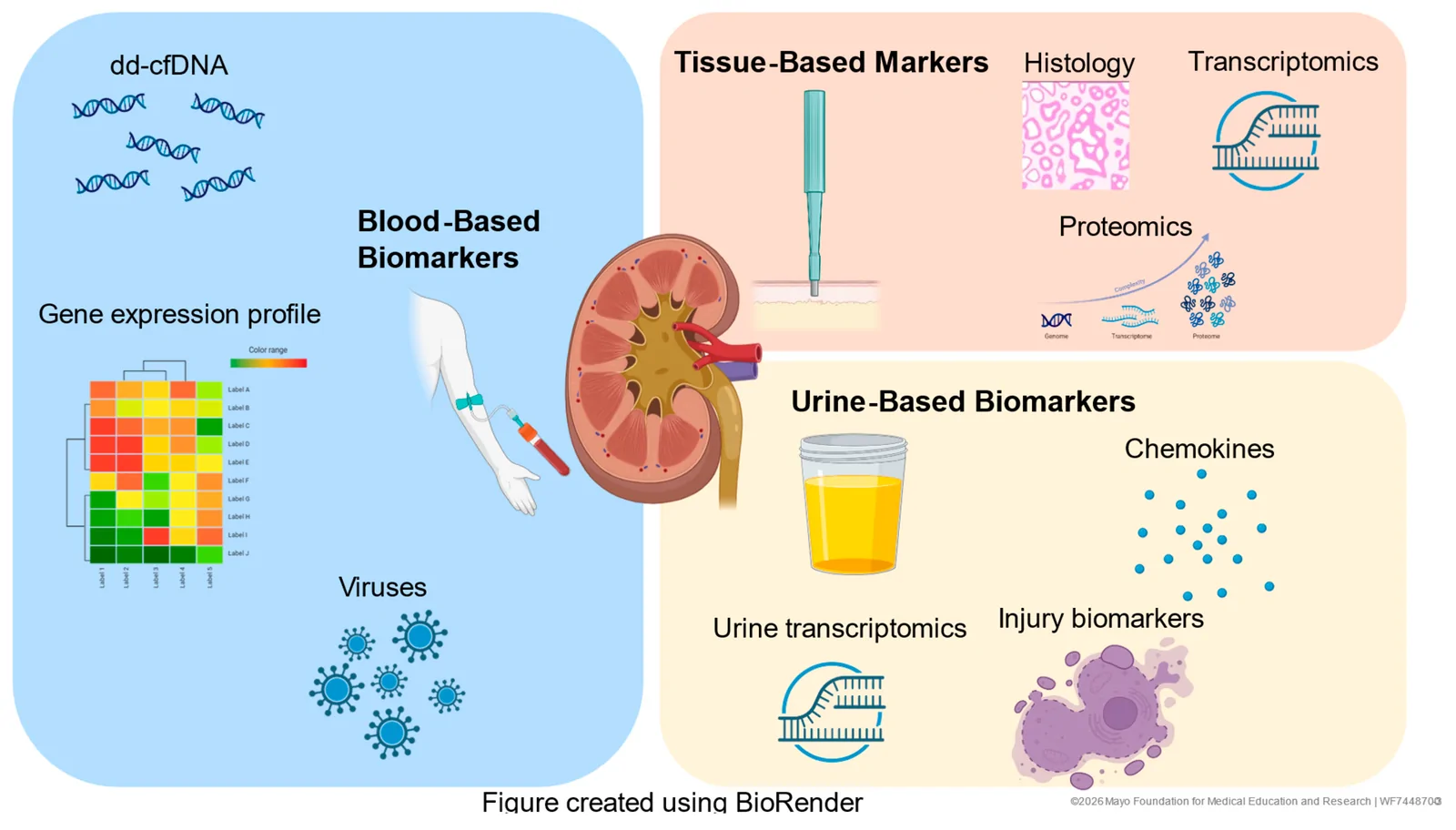
Current landscape of biomarkers: blood-based, urine-based and tissue-based tests. Blood-based biomarkers are minimally invasive and include dd-cfDNA, gene expression profiling and viruses. Tissue-based biomarkers measure sets of transcriptional signatures to define a disease phenotype. Urine-based biomarkers such as chemokines, cytokines, mRNA and urinary exosomal mRNA, can offer direct insight into the allograft microenvironment.

**Figure 2:**
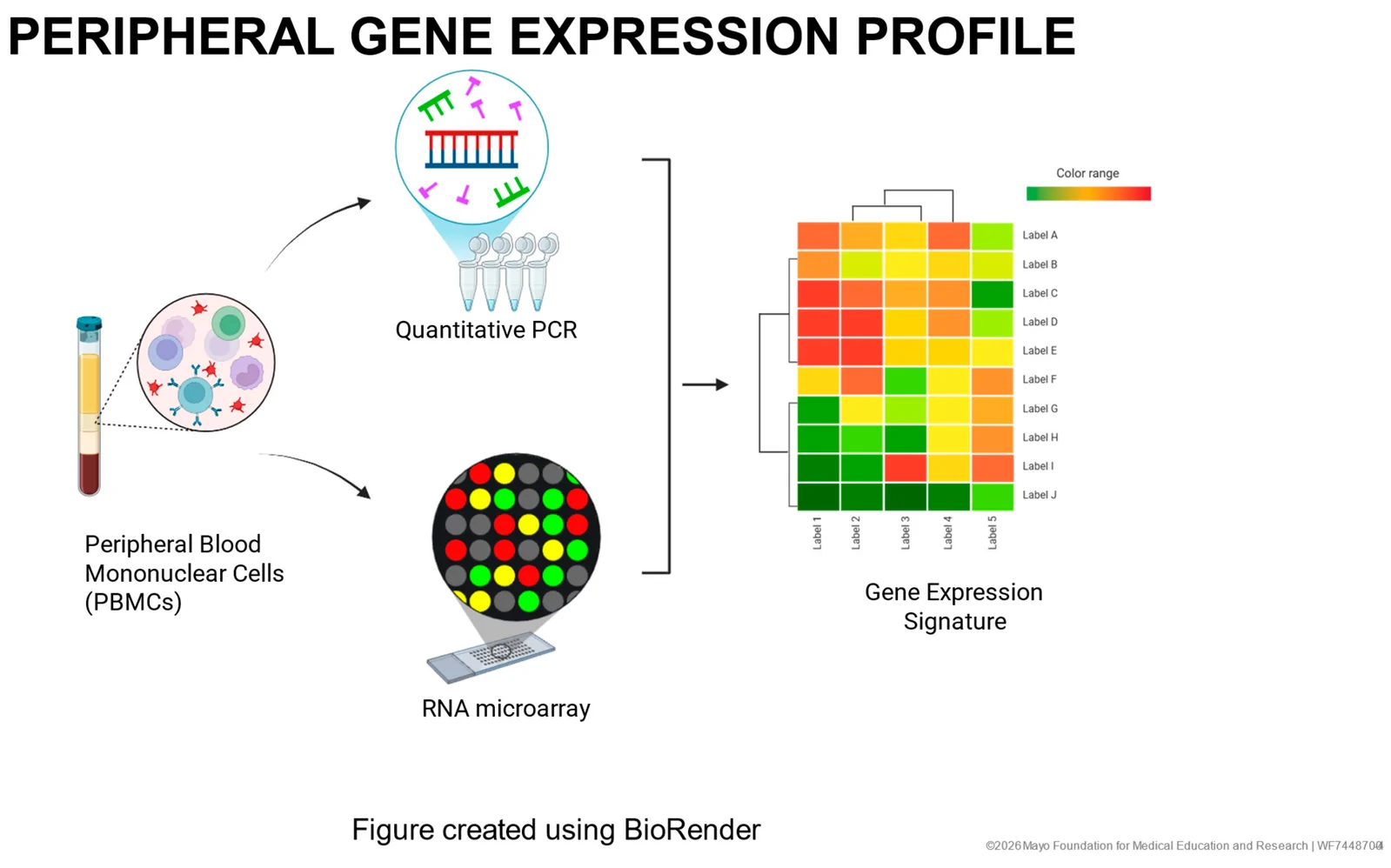
Schematic of the process of gene expression profiling. Peripheral whole blood is collected and PBMCs separated. RNA from a predefined panel of immune response genes is isolated. Quantitative real-time PCR is performed to amplify and quantify target gene expression, which is then used to generate a composite gene expression score.

**Figure 3:**
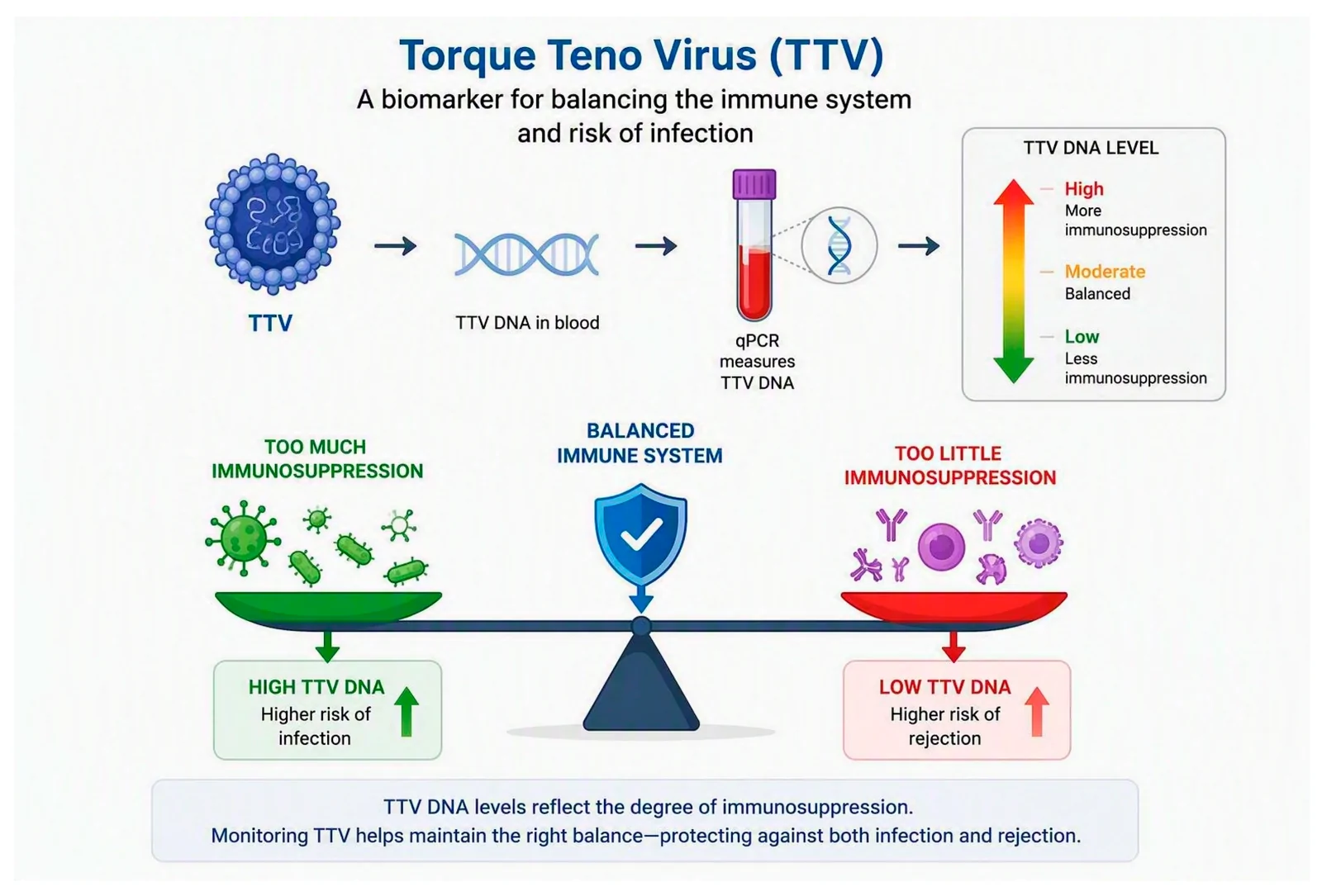
Torque Teno Virus (TTV) as a biomarker of net immunosuppression. High TTV levels are associated with greater immunosuppression and increased risk of infection, whereas low TTV levels may reflect under-immunosuppression and increased rejection risk. Created with Microsoft Copilot based on GPT-5 (AI image generation), August 2026.

**Table 1:** Summary of blood-, urine-, tissue-based biomarkers in kidney transplantation: level of evidence, multicenter validation, key limitations, and clinical readiness.

Biomarker	Sample Type	Evidence Level	Multicenter Validation	Major Limitation	Clinical Readiness
dd-cfDNA	Blood	High	Yes	Cannot distinguish rejection from injury	Implementation is institution dependent
TruGraf^®^ (GEP)	Blood	Moderate-High	Yes	Limited discrimination between AMR and TCMR	Implementation is institution dependent
miRNA	Blood	Low-Moderate		Lack of reproducible signatures	Investigational
TTV	Blood	Moderate	Emerging	Lack of standardized thresholds	Investigational
CXCL9/CXCL10	Urine	Moderate	Yes	Poor specificity, confounded by infection	Investigational/adjunctive
Urinary mRNA	Urine	Moderate-High	Yes	Better for TCMR than AMR	Investigational
Urinary exosomal mRNA	Urine	Moderate	Limited	Technical complexity and cost	Investigational
MMDx™	Tissue	High	Yes	Requires biopsy tissue	Adjunctive clinical use
HistoMAP™	Tissue	Early	Ongoing	Limited validation data	Research

## Data Availability

No new data were created or analyzed in this study. Data sharing is not applicable to this article.
